# Deposition Kinetics of Thin Silica-Like Coatings in a Large Plasma Reactor

**DOI:** 10.3390/ma12193238

**Published:** 2019-10-03

**Authors:** Žiga Gosar, Denis Đonlagić, Simon Pevec, Janez Kovač, Miran Mozetič, Gregor Primc, Alenka Vesel, Rok Zaplotnik

**Affiliations:** 1 Elvez Ltd., Ulica Antona Tomšiča 35, 1294 Višnja Gora, Slovenia; ziga.gosar@elvez.si; 2 Jozef Stefan International Postgraduate School, Jamova cesta 39, 1000 Ljubljana, Slovenia; 3 Faculty of Electrical Engineering and Computer Science, University of Maribor, Koroška Cesta 46, 2000 Maribor, Slovenia; denis.donlagic@um.si (D.Đ.); simon.pevec@um.si (S.P.); 4 Department of Surface Engineering, Jozef Stefan Institute, Jamova cesta 39, 1000 Ljubljana, Slovenia; janez.kovac@ijs.si (J.K.); miran.mozetic@ijs.si (M.M.); gregor.primc@ijs.si (G.P.); rok.zaplotnik@ijs.si (R.Z.); 5 Plasmadis Ltd., Teslova ulica 30, 1000 Ljubljana, Slovenia

**Keywords:** plasma-enhanced chemical vapor deposition (PECVD), hexamethyldisiloxane (HMDSO), industrial-size plasma reactor, capacitively coupled radiofrequency (RF) discharge, ion density, real time deposition rate measurement, time-of-flight secondary ion mass spectrometry (ToF-SIMS)

## Abstract

An industrial size plasma reactor of 5 m^3^ volume was used to study the deposition of silica-like coatings by the plasma-enhanced chemical vapor deposition (PECVD) method. The plasma was sustained by an asymmetrical capacitively coupled radio-frequency discharge at a frequency of 40 kHz and power up to 7 kW. Hexamethyldisilioxane (HMDSO) was introduced continuously at different flows of up to 200 sccm upon pumping with a combination of roots and rotary pumps at an effective pumping speed between 25 and 70 L/s to enable suitable gas residence time in the plasma reactor. The deposition rate and ion density were measured continuously during the plasma process. Both parameters were almost perfectly constant with time, and the deposition rate increased linearly in the range of HMDSO flows from 25 to 160 sccm. The plasma density was of the order of 10^14^ m^−3^, indicating an extremely low ionization fraction which decreased with increasing flow from approximately 2 × 10^−7^ to 6 × 10^−8^. The correlations between the processing parameters and the properties of deposited films are drawn and discussed.

## 1. Introduction

A widely used method for deposition of thin coatings onto solid materials at low temperatures is plasma-enhanced chemical vapor deposition (PECVD). A precursor is leaked into a plasma reactor where it is partially ionized and dissociated, and the resulting species stick to a surface to form a thin and often compact coating. Various precursors can be used to deposit films of appropriate properties. Organosilicon precursors are suitable for deposition of coatings containing silica. Depending on plasma parameters, films of various properties are deposited from almost pure silicon dioxide to a polymer-like coating resembling polydimethylsiloxane. The following literature survey presents important results obtained by various authors using various discharge configurations.

The deposition of silicon dioxide coatings was elaborated by Bourreau as early as 1991 using both hexamethyldisilioxane (HMDSO) and tetraethoxysilane (TEOS) [1]. They found significant changes in deposition kinetics at elevated gas and/or substrate temperatures. Kashiwagi et al. applied HMDSO and oxygen at the pressure inside an experimental plasma reactor of approximately 10 Pa to obtain various coatings from almost pure silicon dioxide to a coating resembling polydimethylsiloxane (PDMSO) [2]. They also used other precursors such as tetramethyl-1,3-bis(chloromethyl)-disiloxane (CMDSO), tetramethyl-1,3-bis(hydroxylbutyl)-disiloxane (HBDSO), and tetramethyl- 1,3-bis(amino propyl)disiloxane (APDSO). They sustained plasma by an inductively coupled radio-frequency (RF) discharge and studied the composition and structure of deposited films using various surface-sensitive techniques. Ropcke et al. [3] used three different discharges for plasma sustenance in experimental reactors, i.e., a planar microwave (MW) plasma source operating at the standard MW frequency of 2.45 GHz, an RF planar reactor at 13.56 MHz, and an electrodeless capacitive coupled RF discharge at 460 KHz. Argon was used as the main gas with an admixture of HMDSO and helium. All sources were characterized by various techniques to determine the rotational and optical excitation temperatures as well as the behavior of various plasma radicals at different discharge conditions. Spatially resolved optical emission characterization enabled observation of large gradients in those plasma reactors, in particular the glass reactor powered with an electrodeless capacitively coupled plasma (CCP). However, the standard CCP revealed a huge H-atom temperature of approximately 2000 K next to the powered electrode, indicating importance the of the gas excitation within and next to the sheath. The H-atom temperature was below 500 K at a distance of a couple of centimeters from the powered electrode. In contrast, Theil et al. reported experiments at room gas kinetic temperature, neglecting any temperature gradients [4], using mixtures of oxygen and HMDSO. They found a gradual decrease of the carbon content in deposited Si_x_O_y_ films with an increasing concentration of oxygen in the gas mixture. The mechanisms of plasma polymerization of various silico-organic monomers were reported by Rau et al. [5]. Apart from HMDSO, they also used divinyltetramethyl disiloxane (DVTMDSO), and octamethyl cyclotetrasiloxane (OMCATS) to deposit films of different structure. Argon was added to gaseous plasma in order to enhance the dissociation of precursors, similar to Ropcke [3]. They reported that the decisive parameters determining the polymer growth rates were plasma power and the monomer evaporation temperature. Unlike other precursors, the DVTMDSO also enabled polymerization under remote conditions. The influence of substrate temperature on the growth kinetic was studied by Sahli et al. [6]. They found the deposition rate decrease with increasing oxygen concentrations in a broad range of temperatures up to approximately 400 °C and reported decreasing carbon concentrations in the films with increasing oxygen concentration in the gas mixture. Korzec et al. [7] compared direct and remote plasma deposition of coatings using HMDSO admixed with argon and oxygen. Microwave discharge was used as a plasma source. The remote technique was found favorable because it prevented otherwise unwanted deposition of coatings on reactor walls, as reported by Korzec. A similar device was also used by Tierich et al. [8] except that they used a remote RF source. A remote microwave plasma was also used by Bieder et al. [9]. Kurunczi et al. [10] also used tetramethylsilane (TMS) and measured optical spectra arising from excited gaseous species upon excitation with electrons of various kinetic energy. The extensive experimental work brought the conclusion that the single-step dissociative excitation processes were unlikely to play a dominant role in the low-temperature processing plasma of these precursors because the excitation thresholds were large compared to typical electron temperatures in plasma reactors. Alexander et al. [11] characterized plasma sustained in HMDSO also by mass spectrometry. Extensive molecular oligomerization of positively charged gaseous species was observed in the plasma gas phase at a rather low discharge power, whereas at large powers, fragmentation prevailed, similar to what was reported by Ropcke [3]. Interestingly enough, the oligomerization was not observed for neutral gaseous species irrespective of the discharge power. They concluded that ion-molecule reactions were responsible for deposit-formation at a low discharge power, but at a high power, the ionic component became important. Fujii et al. [12] investigated the composition of thin films prepared by plasma polymerization from HMDSO with an admixture of fluorine-containing gases to obtain deposits of low dielectric constant. They applied remote inductively coupled plasma as a radical source. Similar results were reported by Borvon et al. [13] except that they admixed methane to a HMDSO-O_2_ gas mixture. Shioya et al. [14] also reported low-k dielectric films, but they used HMDSO admixed with water vapor. Gengenbach et al. [15] also used hexamethyldisilazane (HMDSA) apart from HMDSO and studied the ageing mechanisms stressing the post-deposition kinetics. Hagemann [16], on the other hand, used a mixture of HMDSO and oxygen to obtain coatings of various silicon-to-carbon ratios. In another paper [17], they reported results of experiments obtained using both symmetric and asymmetric discharges and found the crucial correlation between the gas flow and the discharge power on the deposition rate. An interesting electrode configuration was reported by Nema et al. [18], who reported the excellent quality of films deposited using HMDSO and oxygen precursors. Zywitzki et al. [19] used other types of discharges, i.e., reactive pulsed magnetron sputtering and hollow cathode activated electron beam evaporation, for plasma deposition of coatings from HMDSO. They reported extremely high deposition rates up to 600 nm/s using these powerful discharges. Qi et al. [20] reported much smaller deposition rates using microwave discharge where the electron cyclotron resonance (ECR) conditions were obtained. The deposition rates were several tens of nanometers per second. One of the highest deposition rates was observed by using a plasma micro-jet as reported by Silmy et al. [21]. They reported deposition rates as large as 0.1 mm/s. Fang et al. [22] applied HMDSO admixed with various gases and studied the deposition rate in inductively coupled RF plasma at the same discharge conditions and found the lowest rate for the case of the hydrogen admixture and highest for oxygen. Xiao et al. [23] admixed ammonia to HMDSO and observed a solid growth of a coating resembling silicon nitride of excellent hardness. Ammonia was also admixed to HMDSO and hydrocarbons by Hegemann et al. [24]. Vautrin et al. [25] studied the influence of substrate biasing and observed that RF bias induced a densification of the coatings associated with modifications in their morphology and chemical composition. Lee et al. performed deposition of PDMS using PDMS/O_2_/He mixture [26]. The deposition rate increased with increasing PDMS flow rate as well as surface roughness. The deposition rate also increased with increasing O_2_ flow rate; however, surface roughness decreased. At some certain conditions, SiO_2_-like coatings were obtained with a deposition rate of 12 nm/min.

All authors cited above used low-pressure experimental plasma reactors. A somehow elevated HMDSO pressure of 133 Pa was used by Goujon et al. [27], who also applied moderate discharge power to sustain plasma in HMDSO-oxygen mixture using a capacitively coupled RF discharge to obtain strong fragmentation of the precursor.

The brief review of relevant literature reveals a variety of discharges used for deposition of silica-like coatings. The useful pressure upon plasma polymerization was in the range of few Pascals. The advantage of low pressure discharges is the ability to sustain rather uniform plasma in a large volume and at a reasonably low discharge power density. In fact, most current commercial devices for deposition of such thin films on solid substrates employ low pressure reactors to insure reasonable uniformity and stoichiometry of the coatings and to prevent formation of dust particles in the gas phase. Such particles are detrimental for protective coatings because they cause the formation of porous films of poor quality, as explained by Gosar et al. [28].

Gaseous plasma created at low pressure conditions was characterized by various techniques, but only few papers report quantitative results such as the density of charged particles, let alone the densities of molecular fragments. Plasma density is usually measured with an electrical probe. The probe is immersed into an experimental reactor and its characteristics (current versus voltage) is measured. The charged particle density is then deduced using an appropriate model. The calculation requires known ion mass (if ion saturated current is used for calculation of the plasma density), which is generally not available because the precursor dissociates to various fragments upon plasma conditions, and the fragments ionize at various rates. Furthermore, any electrode is quickly covered with a dielectric film, which complicates the interpretation of results. Particularly difficult is determination of charged particle density in industrial reactors where it is often difficult if not impossible to mount a suitable probe inside the reactor.

The properties of thin films are typically characterized with a remote instrument that enables determination of the film composition and/or structure, such as X-ray photoelectron spectroscopy (XPS) or secondary ion mass spectrometry (SIMS) depth profilers. Very few authors ever attempted to measure the deposition rate in situ due to the lack of appropriate methods. It is particularly difficult to adopt a standard technique for measuring the deposition rate into an industrial reactor, so the users of PECVD technology for deposition of silica-like coatings from organometallic precursors do not have a reliable method for controlling the deposition rate upon treatment of their products in large reactors. In the present paper we report such a method and discuss the deposition rates in the view of plasma parameters.

## 2. Materials and Methods

An industrial-size plasma reactor was used to study the deposition of silica-like films onto polymeric substrates. The system has been presented in detail elsewhere [28]. The discharge chamber of a volume of approximately 5 m^3^ was powered with an RF generator at a frequency of 40 kHz using an asymmetrical capacitively coupled RF discharge. The powered electrode had an area of 0.8 m^2^, whereas the total surface of the grounded housing was approximately 15 m^2^. HMDSO was introduced into the reactor through numerous small holes, thus assuring for a rather uniform distribution of the precursor leaked into the reactor. The HMDSO flow was measured with a flow-meter calibrated for this gas. The pressure was measured inside the reaction chamber with a Pirani gauge. The reactor was pumped through connecting tubes of various diameters separated by valves with a roots blower with a nominal pumping speed of 8800 m^3^/h backed with a rotational pump of pumping speed 1260 m^3^/h. The measured gas flow and pressure enabled for estimation of an effective pumping speed and thus the gas residence time inside the plasma reactor. Several samples made from stainless steel were carefully polished and mounted into the plasma reactor. The deposition of thin films resembling polydimethylsiloxane was accomplished at a discharge power of 3.4 kW and a HMDSO flow of 130 sccm.

The deposition rate of the coating was measured with a homemade optical sensor. The optical sensor consists of an all-fiber Fabry-Perot resonator that is formed at the tip of an optical fiber. More detailed information about the sensor and its construction has been presented elsewhere [29,30]. The sensor measures the thickness of the deposited layer every second in a resolution below 1 nm. At certain plasma conditions, the thickness of the coating was measured for approximately 100 s. From the slope of the thickness-to-time evolution curve, a deposition rate was determined.

The coating thickness of one selected sample was analyzed by scanning electron microscope (SEM). The JSM-5800 microscope from Jeol (Scan d.o.o., Ljubljana, Slovenia) was used. To determine the coating thickness, a cross-section of the sample was prepared. The procedure for the preparation of the cross-section included inserting the sample into a special powder that was sintered at a high temperature and then polished to remove a part of the material. It should be noted that this procedure can cause some irregularities in the uniformity of the coating.

Moreover, the coating thickness and composition was also examined by time-of-flight secondary ion mass spectrometry (ToF-SIMS). The ToF–SIMS 5 instrument (ION-TOF, Münster, Germany) equipped with a bismuth liquid metal ion gun with a kinetic energy of 30 keV was used. The SIMS spectra were measured by scanning a Bi_3_^+^ cluster ion beam over an area of 100 × 100 µm^2^. The beam current was 0.6 pA, and the total measuring time to acquire the SIMS spectra was 30 s. The SIMS spectra were processed with the software SurfaceLab 6.3 supplied by ION TOF (Münster, Germany).

Surface chemical composition was also investigated using X-ray photoelectron spectroscopy (XPS). The PHI-TFA XPS spectrometer (Physical Electronics, München, Germany) was used. Samples were exposed to X-ray radiation from monochromatic Al source with a photon energy of 1486.6 eV. High resolution spectra were measured at a pass energy of 29.35 eV and 0.125 eV energy step for the deposited coatings and also for the reference samples of pure PDMS and SiO_2_. An additional electron gun was used during spectra acquisition for surface charge compensation. All spectra were referenced to the main C1s peak of the carbon atoms C-Si, which was assigned a value of 284.4 eV [31]. Quantification of the surface composition was performed using Multipak v8.1c software (Ulvac-Phi Inc., Kanagawa, Japan, 2006), which was supplied with the spectrometer. The spectra were fitted with Gauss-Lorentz functions. A Shirley type background subtraction was used. The FWHM was fixed during the curve fitting process (1.2 eV for C1s and 1.4 eV for O1s and Si2p). First, the reference samples of PDMS and SiO_2_ were fitted to obtain the best fitting parameters, which were used for further fitting of spectra recorded for the deposited coatings.

## 3. Results and Discussion

**Figure 1:**
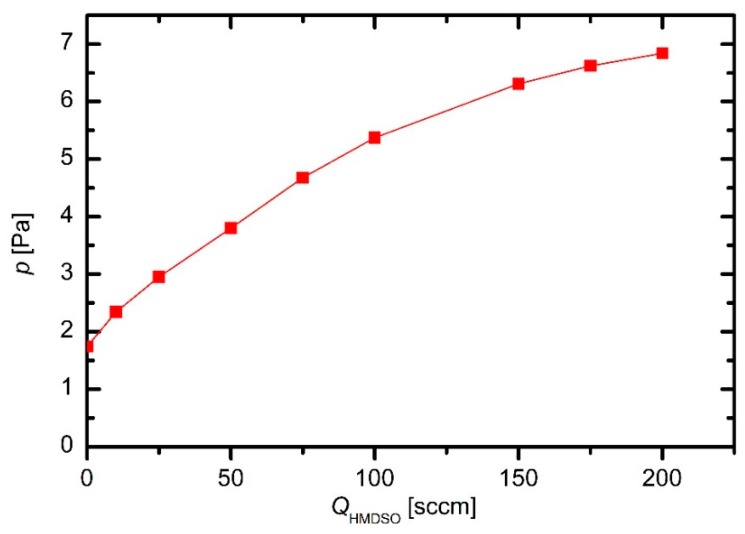
The pressure in the plasma reactor versus the hexamethyldisilioxane (HMDSO) flow.

The pressure in the plasma reactor was measured versus the flow of HMDSO when the discharge was off. The pressure dependence is plotted in Figure 1. The pressure at zero flow of HMDSO represents the residual atmosphere. Because the system was hermetically tight, the finite pressure at zero flow is attributed to desorption of gases, in particular water vapor, from the surface of the materials mounted into the plasma reactor. Although a Pirani gauge is not the most accurate device for measuring the pressure in the range of few Pascals, Figure 1 indicates a rather large density of water molecules in the plasma reactor. As expected, the pressure increases with the increasing flow of HMDSO, but the curve in Figure 1 is not linear.

The deviation from linearity in Figure 1 is explained by the pressure-dependent effective pumping speed. Because the discharge was off, there was no loss of the precursors, which otherwise occurs upon plasma conditions because a fraction of precursor is spent for growing the films. The device was hermetically tight, therefore the mass flow through the system was constant at a given leakage of HMDSO and the continuity equation is applicable:

*p*_1_*S*_1_ = *p*_2_*S*_2_ (1)

Here, *S*_x_ (x = 1, 2) is the effective pumping speed (the volume flow) at the position where pressure *p*_x_ is measured. The pressure at the flow controller where the flow is measured is by definition 1 bar, i.e., *p*_2_ = 1 bar, whereas *S*_2_ is the flow measured with the flowmeter. The pressure *p*_1_ is measured in the plasma reactor, hence the effective volume flow through the reactor is

*S*_1_ = *p*_2_*S*_2_/*p*_1_ (2)

**Figure 2:**
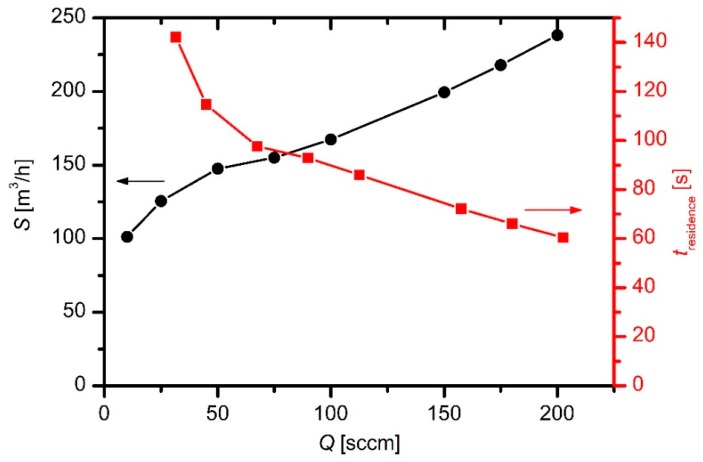
The effective pumping speed and the gas residence time in the plasma reactor versus the HMDSO flow.

It is not feasible to calculate the effective pumping speed from the values represented by points in Figure 1, therefore Equation (2) should be modified to consider continuous desorption of water vapor:

*S*_1_ = *p*_2_*S*_2_/(*p*_1_ − *p*_res_) (3)

where *p*_res_ is the pressure of the residual atmosphere. The effective pumping speed versus the HMDSO as determined from Equation (3) is shown in Figure 2. It increases with increasing flow, which is explained by two effects: increasing compression rate of the roots pump in the pressure range of several Pascals with increasing pressure, and the finite conductivity of the vacuum components between the plasma reactor and the roots pump. The effective pumping speed is much smaller than the nominal pumping speed of the roots pump. As stated in the previous section, the nominal pumping speed of the roots pump was 8800 m^3^/h at optimal pressure and fore-pumping.

The residence time of the gas leaked into the plasma reactor is estimated from the effective pumping speed and the cross-section of the reactor:

*t* = (*l A*)/*S*_1_ (4)

where *l* is the length of the plasma reactor (i.e., distance between the gas inlet and the pumping duct), *A* is the cross-section of the plasma reactor, and *S*_1_ is the effective pumping speed calculated from Equation (3). Taking into account numerical values, i.e., *l* ≈ 2 m, with *A* ≈ 2 m^2^ the residence time is of the order of 100 s. The exact values are plotted in Figure 2 (right *y*-axis). The residence time is therefore large enough to assure the decent fragmentation of the HMDSO upon plasma conditions.

The plasma reactor does not allow for insertion of a probe for measuring plasma density, thus this parameter is estimated taking into account some simplifications. The plasma inside the reactor is assumed to be rather homogeneous, with comparable densities of free electrons and positively charged ions. The electrons are rather hot in low-pressure non-equilibrium gaseous plasma sustained by a discharge of a low power density with a typical temperature of a few electronvolts. The positive ions are thermal at the temperature similar to the neutral gas kinetic temperature, which in turn is close to the wall temperature. While the powered electrode is heated to several tens of kelvins above room temperature upon continuous discharge, the temperature of the chamber walls remains almost at room temperature even for prolonged plasma duration; therefore, it can be assumed that the ions in bulk plasma are close to the room temperature, i.e., 300 K. The ions are accelerated in the pre-sheath next to electrodes, gaining directed velocity, but their concentration in the pre-sheath remains almost the same as in bulk plasma. According to classical literature [32], the ions gain Bohm velocity across the pre-sheath:

(5) $$v_{Bohm}=\sqrt{\left(kTe\right)/M)}$$

where *k* is Boltzmann constant, *T*_e_ is the electron temperature in bulk plasma, and *M* is the ion mass. The ions therefore enter the sheath next to electrodes with the velocity as in Equation (5). The ions are accelerated in the potential drop next to the electrodes. In the case of capacitively coupled RF discharges, the self-biasing of the electrodes occurs. In our case, the discharge is heavily asymmetrical with the ratio of the powered and grounded electrode areas at almost two orders of magnitude. Because the RF self-biasing is roughly proportional to the square of the area ratio, it can be assumed that practically all DC voltage due to electrode self-biasing occurs on the powered electrode. The ions are accelerated in the time-averaged voltage across the sheath and bombard the electrode with the kinetic energy of

**Figure 3:**
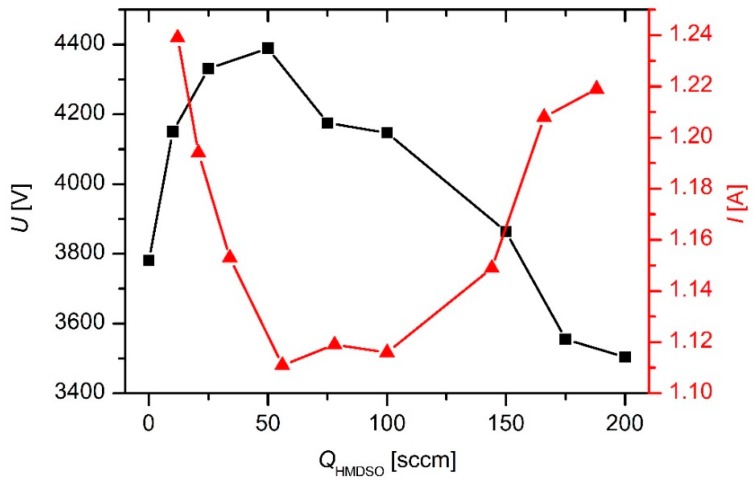
The voltage (left-hand *y*-axis) and current (right-hand *y*-axis) versus the HMDSO flow.

*W_kin_* = *e U*, (6)

where *e* is the ion charge, and *U* is the time-averaged voltage across the sheath next to the powered electrode (i.e., the DC voltage due to self-biasing). Practically all ions in weakly ionized plasma sustained by a discharge of a low power density are singly-ionized, so *e* from Equation (6) is substituted with the elementary charge, i.e., *e* = *e*_0_ = 1.6 × 10^−19^ As. In a rough approximation, the voltage drop *U* can be replaced with the effective voltage of the power supply. There are voltage and current meters on the power supply. The voltage and current versus the HMDSO flow are plotted in Figure 3.

The average velocity of the ions bombarding the powered electrode is obtained from its kinetic energy and Equation (6):

(7) $$v=\sqrt{2W_{kin}/M)}=\sqrt{2e_{0}U/M)}$$

As in Equation (5), *M* is the ion mass. There are ions of various masses from H_2_^+^ to C_6_H_18_OSi_2_^+^, so the choice of velocities in Equation (7) is somehow arbitrary. Furthermore, Alexander et al. [11] reported oligomerization of positively charged ions. Still, in a very rough approximation, the velocity *v* can be attributed to the mass of the most common molecular fragments. According to Gosar et al. [28], who performed measurements of the fragments in this particular plasma reactor by mass spectroscopy, the densest fragments are the positively charged ions of a mass of approximately 30.

The flux (*j*) of ions onto the powered electrode is a product of the ion density (*n*_+_) next to the electrode and the “most probable” velocity (v), i.e., *j* = *n_+_v*, thus the ion current (*I*) is

**Figure 4:**
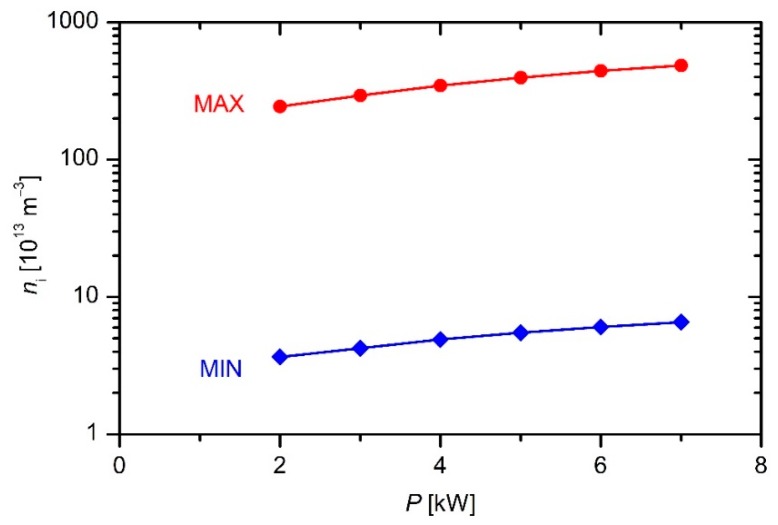
The limits of the plasma density versus the discharge power at an HMDSO flow of 130 sccm.

**Figure 5:**
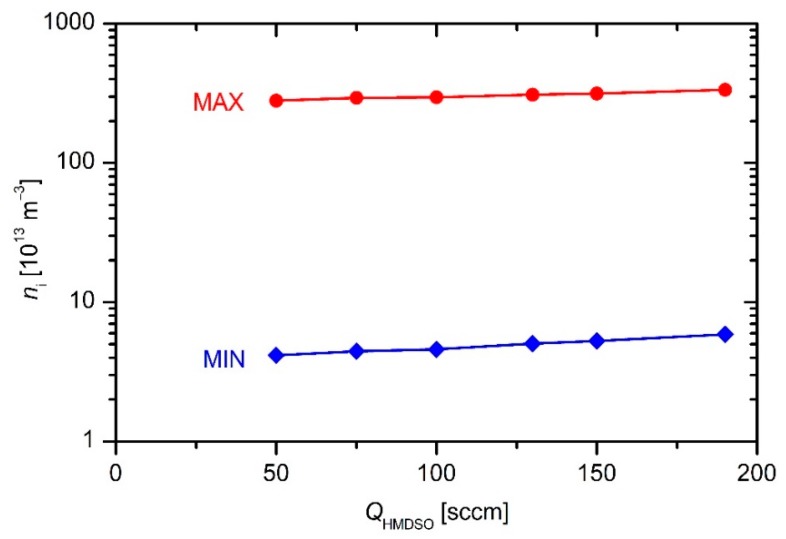
The limits of the plasma density in the industrial reactor versus the HMDSO flow at a discharge power of 3.4 kW.

*I* = *e_0_ j A_el_* = *e_0_ n_+_ v A_el_* (8)

where *n*_+_ is the density of positive ions next to the powered electrode, and *A*_el_ is the geometrical area of the powered electrode. The Equation (8) makes a reasonable approximation if the electrical current next to the electrode is conducted by ions only. In practice, it never happens for at least two reasons: (1) additional current due to electrons emitted from the powered electrode by ion bombardment; and (2) the effects within the short time interval when the voltage on the powered electrode is at its maximum. Still, Equation (8) gives a useful approximation of the ion density deep in the sheath next to the electrode. The ion density in plasma far away (tens or even hundreds of Debye lengths away) from the powered electrode is larger, and therefore the value of the plasma density as determined from Equation (8) can be taken only as the lower limit. This lower limit is plotted in Figure 4 and Figure 5 versus the discharge power and precursor flow, respectively.

The upper limit of the plasma density can be stated by considering the conditions at the interface of the sheath and pre-sheath. The ion velocity at this interface is determined from Equation (5). If the ions were the major current carriers at the interface, Equation (8) would have been applicable for the estimation of the ion density in gaseous plasma just by replacing the ion velocity *v* in Equation (8) with the Bohm velocity as in Equation (5):

*n* = *I*/(*e*_0_*v*_Bomh_*A*_el_) (9)

Such an assumption, however, is not justified due to the much larger mobility of electrons as compared to massive ions. Despite the potential drop between the unperturbed plasma and the interface between the pre-sheath and the sheath, the electrical current is predominantly conducted by electrons because of their much larger mobility, thus any calculation of the plasma density using Equation (9) will give an overestimated value. Still, Equation (9) is useful for estimation of the upper limit of the ion density in a plasma reactor where adoption of a probe is not feasible. The upper limits of the plasma density as calculated from Equation (9) are plotted in Figure 4 and Figure 5. The numerical values of plasma density also requires the electron temperature that is generally unknown. Happily, the electron temperature in any plasma sustained by an asymmetric RF discharge at the pressure of few Pascals in a molecular gas is roughly a few electronvolts. The Bohm velocity depends on the square of the electron temperature, and therefore any mistake because of the wrong *T*_e_ is marginal as compared to other simplifications taken into account to estimate the upper value of the plasma density.

Again, it should be stressed that the values obtained from Equation (9) are highly overestimated because the electron contribution to the electrical current as measured for the electrical circuit used for sustaining plasma in our industrial reactor is higher than the ion current. On the other hand, the values obtained using Equation (8) are highly underestimated because they do not take into account the gradient of the ion density across the sheath. Fortunately, the upper and lower limits of the ion density are not too far away from each other as revealed from Figure 4 and Figure 5, and therefore they represent useful data in the reactor where an electrical probe could not be mounted.

The results of such simplifications only give the order of magnitude. Obviously, the plasma density in the reactor is of the order of 10^14^ m^−3^. Examining both the upper and lower limit one can deduce a rather weak increase of the plasma density with increasing discharge power or flow rate.

**Figure 6:**
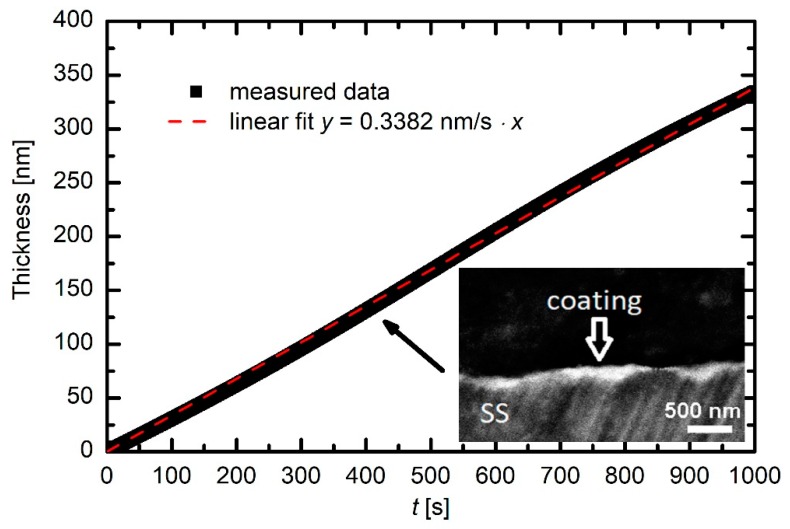
The film thickness versus deposition time. Inset shows a scanning electron microscope (SEM) image of a cross-section of a selected sample after deposition for approximately 400 s.

Plasma density influences the gas-phase reactions and, correspondingly, the deposition of coatings containing carbon, hydrogen, silicon, and oxygen on the surfaces of substrates facing plasma. To investigate the deposition kinetics, we immersed several optical probes for real-time measuring of the deposition rate. Details of the probes are given in Section 2. Typical deposition kinetics are revealed in Figure 6. The thickness of the deposited film increases almost perfectly linearly with increasing deposition time, indicating very stable conditions. The coating thickness was also confirmed by SEM analysis of a cross-section of one selected sample. This image is shown as an inset in Figure 6. Although its resolution is not the best because of using low-resolution SEM microscopy and because of the procedure for the preparation of the cross-section (see Section 2), it is nevertheless clear that the coating thickness is in agreement with the measured curve.

**Figure 7:**
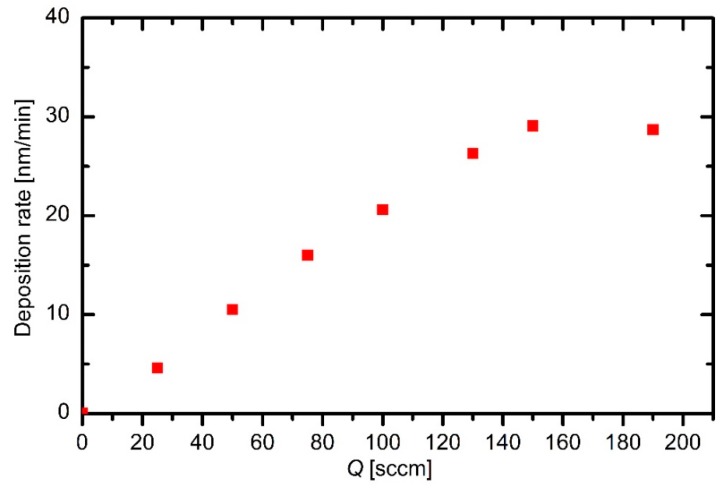
Deposition rates versus HMDSO flow at a discharge power of 3.4 kW.

The slope of the curve in Figure 6 allows for determination of the deposition rate. The rates measured at different HMDSO flows are shown in Figure 7. One can observe an almost linear increase of the deposition rate with increasing precursor flow. Compared to the behavior of plasma density (Figure 5), the slope of the deposition curve is much larger than the slope of the plasma density. The discrepancy is attributed to the fact that the major building units of the deposited films are not positively charged, but they are rather neutral reactive particles. In fact, the deposition rate is of the order of 10 nm/min, which is much larger than the maximal deposition rate calculated from the estimated ion flux onto the sample surface using results presented in Figure 4 and Figure 5. This observation is different from results reported for the case of rather small experimental reactors. For example, Ropcke [3] reported strong gradients in radical emissions that in turn caused spatial dependence of the deposition rate. The deposition rate correlated well with the intensity of optical emission. They used a capacitive coupled electrodeless RF discharge for sustaining plasma in a tube of a volume of approximately 50 cm^3^ and a discharge power of a few tens of watts, and therefore the power density was of the order of 10^5^ W/m^3^. Furthermore, they used argon as a major gas with a small admixture of HMDSO. At such conditions, the precursor was highly dissociated already upon entering the gas into the plasma zone, and therefore the thickness of the deposited film decreased with increasing distance from the injection point. In the electrodeless CCP configuration, no significant change of the electron temperature (as derived from the H_α_/H_β_ ratio) was detected, although the gas composition changed significantly along the glass tube. This observation indicated the importance of the argon/hydrogen kinetics at a relatively low level of HMDSO.

The deposition rate at the highest flow deviates significantly from the general trend (Figure 7), although the ion density (Figure 5) keeps increasing with increasing HMDSO flow in the entire range of flows. The discrepancy can be explained by the fact that the ion density was deduced from the measured electrical current on the powered electrode, whereas the thickness sensor was mounted far from the electrodes. In one configuration, Ropcke [3] employed a standard capacitive RF discharge and observed a huge gradient in the hydrogen atom kinetic temperature (as deduced from H_α_ line broadening) next to the powered electrode. This observation indicates important differences between the sheath and plasma kinetics in capacitive RF discharges. The tendency toward a saturation of the deposition rate at higher HMDSO ratios in plasma far away from the powered electrode may be a consequence of the more extensive collisions next to the powered electrode and thus unwanted film deposition on the electrode on the expense of a film deposited on the surface of any other material mounted into the plasma reactor.

**Figure 8:**
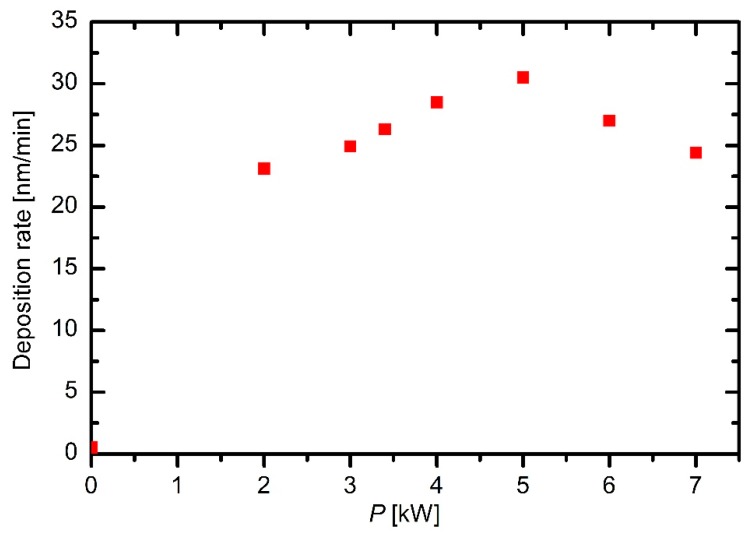
Deposition rates versus discharge power at an HMDSO flow of 130 sccm.

The deposition rate versus the discharge power exhibits a more interesting behavior. A maximum at a power of approximately 5 kW is observed in Figure 8. Though these measurements were performed only at the flow rate of 130 sccm, and thus they cannot be regarded a general trend, they indicate rather interesting deposition kinetics. Here, it is worth recalling the densities of particles in the plasma reactor. The plasma density is of the order of 10^14^ m^−3^ as revealed from Figure 4 and Figure 5, whereas the density of neutral gaseous molecules is of the order of 10^21^ m^−3^. The ionization rate is therefore only of the order of 10^−7^. The original HMDSO molecules will not contribute to the growth rate, but some of their fragments would. A larger discharge power results in the larger plasma density and thus more intensive fragmentation of the precursor molecules. Not all fragments, however, are suitable as the building units for the deposit. Furthermore, a larger power represents a higher thermal load for the powered electrode, which in turn causes higher temperatures and thus preferential deposition of the coating on the powered electrodes. In fact, Ropcke et al. [3] reported strong gradients in plasma parameters at elevated power densities causing an interesting behavior of deposition kinetics.

In any case, the deposition rate is of the order of 10 nm/min (see Figure 7 and Figure 8). At this point, it should be stressed that these deposition rates were measured rather near the electrodes, and therefore, these values are the upper limit of the deposition rates inside the reactor. Further away from the electrodes, the deposition rates are smaller. The flow in Figure 8 was set at 130 sccm. The mass flow (*Φ_mas_*) through the system is calculated as the product of the volume flow (*S*_2_) and mass density (*ρ*) at the pressure where the volume flow is measured (*Φ_mass_ =* S*_2_* *ρ*). The mass density (*ρ*) of gas is calculated from the number density and the mass of the molecule taking into account an ideal gas law, and therefore the mass flow is

*Φ_mass_* = S*_2_ρ* = *S_2_ M_HMDSO_ p_2_/(kT)* (10)

where *M*_HMDSO_ is the mass of a HMDSO molecule (i.e., 2.8 × 10^−25^ kg), *S*_2_ is the volume flow as determined by the flowmeter, and *p*_2_ is the pressure at the flow meter, i.e., 1 bar. The mass flow through the system at the flow rate of 130 sccm (= 2.17 × 10^−6^ m^3^/s) is therefore approximately 1.5 × 10^−5^ kg/s. At these conditions, the measured deposition rate is approximately 25 nm/min (see Figure 8). The mass deposited in unit time on the surfaces of any components placed in the plasma reactor is calculated from the measured deposition rate and the estimated density of the deposit and total surface area of any materials facing plasma:

d*m*/d*t* = d*x*/d*t ρ_dep_ A_tot_* (11)

The parameters in Equation (11) can only be roughly estimated. Taking into account the density of the PDMSO-like coating *ρ*_dep_ = 10^3^ kg/m^3^, the total surface area *A*_tot_ = 15 m^2^, and the measured deposition rate d*x*/d*t* = 25 nm/min, one gets the quantity of mass deposited on the surface of the reactor in unit time d*m*/d*t* = 0.6 × 10^−5^ kg/s. This value is surprisingly close to the mass flow as calculated from Equation (10). However, this value again is the upper limit due to the fact that the upper limit deposition rate, measured near the electrodes, is used for this calculation. The average deposition rate throughout the reactor is probably almost an order of magnitude lower, and consequently, the d*m*/d*t* is lower.

**Figure 9:**
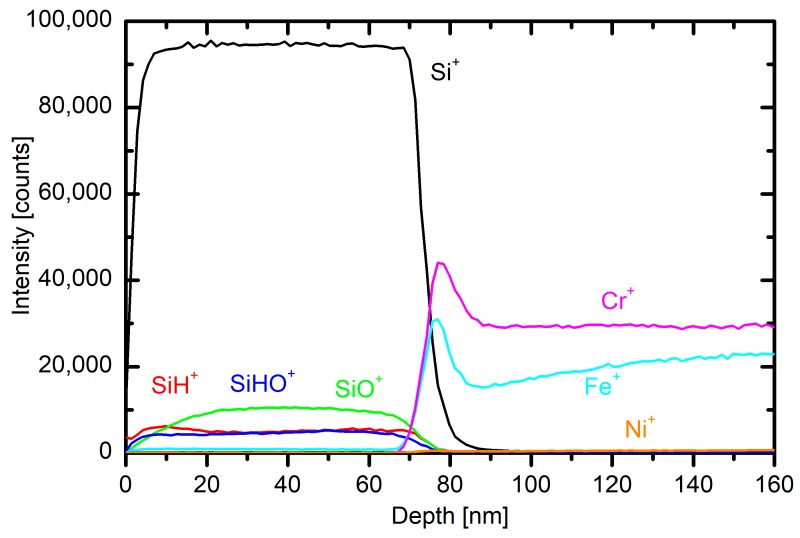
A typical secondary ion mass spectrometry (SIMS) depth profile of the polydimethylsiloxane (PDMSO)-like coating where only the most intense mass peaks are presented.

Several small stainless steel samples were mounted onto planetaria in the plasma reactor. The samples were kept in the plasma reactor for 20 min at a flow of 130 sccm and 3.4 kW power and then characterized by ToF-SIMS depth profiling. A typical profile is shown in Figure 9. One can recognize a polymer-like coating resembling polydimethylsiloxane.

The concentrations of elements are somehow different from the theoretical value, but SIMS is not the best technique for determination of absolute concentrations of the elements. Still, the depth profile shows a reasonably uniform coating that is useful as a protection coating. The depth profile of the sample, which was positioned on the other side of the reactor furthest from the electrode, also proves that the deposition rate is not uniform across the reactor, and the average deposition rate is smaller than the ones measured near the electrode.

**Figure 10:**
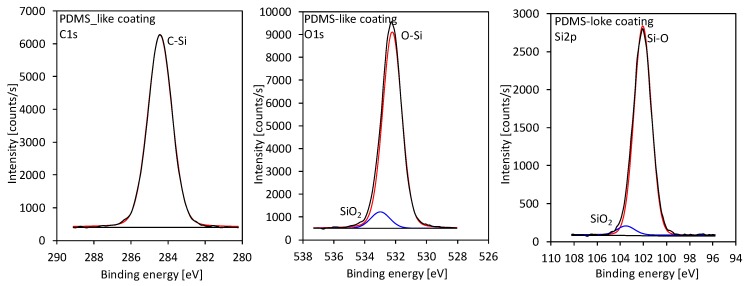
High-resolution X-ray photoelectron spectroscopy (XPS) spectra of carbon C1s, oxygen O1s, and silicon Si2p for the deposited coating.

Additional surface analyses were performed using XPS to reveal a detailed chemical composition of the coating. The surface composition as calculated from the survey spectra showed similar concentrations of oxygen and silicon, i.e., approximately 26 and 25 at%, respectively, whereas the concentration of carbon was approximately 49 at%. Such surface composition of the deposit is very similar to the theoretical composition of PDMS, indicating that PDMS is a dominant component of the coating. Similar results were also found by other authors [33,34]. However, to get more insight into the chemical composition, high-resolution spectra of C1s, Si2p, and O1s were also measured and compared to the spectra measured for the reference samples of pure PDMS and SiO_2_. Deconvolution of high-resolution XPS spectra for C1s, Si2p, and O1s is shown in Figure 10, whereas the comparison of the spectra with the reference samples is shown in supplemental information in Figure S1 (Supplementary Materials). The results showed that the coating consists mostly of PDMS with some presence of a SiO_2_ component. Some additional analysis of the chemical composition of the samples can be also found in our recent paper [28].

## 4. Conclusions

The deposition kinetics for thin films by plasma-enhanced chemical vapor technique using hexamethyldisiloxane in a large industrial plasma reactor was studied. The available discharge parameters were the gas pressure and flow; the pumping speeds of the pumps; the voltage, current, and power of the RF generator used for sustaining an asymmetrical discharge in the capacitive mode; and the geometry of the system. The deposition kinetic was measured by optical interferometry using a custom-designed miniature fiber-optics sensor, whereas the structure of the deposited films was determined after the treatment by SIMS depth profiling. The combination of available data allowed for an insight in the deposition kinetics. The plasma density was roughly estimated from measured discharge parameters because the industrial system did not allow for the immersion of a suitable electrical probe. The ionization fraction was of the order of 10^−7^, but the residence time of gas in the reactor was approximately 100 s, so the gaseous precursor was well-dissociated to various radicals. This enabled the growth of a film of a composition close to polymethyldisiloxane. The available material for the growth of the thin film was rather well utilized because the amount of material as calculated from the measured deposition rate was comparable to the available mass calculated from the flow of gas through the system.

## Supplementary Materials

The following are available online at https://www.mdpi.com/1996-1944/12/19/3238/s1, Figure S1: Comparison of XPS spectra of the coating with the reference samples of PDMS and SiO_2_ for (**a**) C1s, (**b**) O1s, and (**c**) Si2p, and detailed surface spectra of the coating with subcomponents for (**d**) C1s, (**e**) O1s, and (**f**) Si2p.
