# Supplementary material for: Deposition Kinetics of Thin Silica-Like Coatings in a Large Plasma Reactor
Source: Materials (Basel). 2019 Oct 3;12(19):3238. doi: 10.3390/ma12193238 (PMC6803826; doi:10.3390/ma12193238)
Supplement: Supplementary file 1 [file materials-12-03238-s001.pdf]

Materials **2019**, *12*, 1; doi: 10.3390/ma12193238

Supplementary Materials: Deposition Kinetics of Thin Silica-Like Coatings in a Large Plasma Reactor

Žiga Gosar, Denis Đonlagić, Simon Pevec, Janez Kovač, Miran Mozetič, Gregor Primc, Alenka Vesel and Rok Zaplotnik

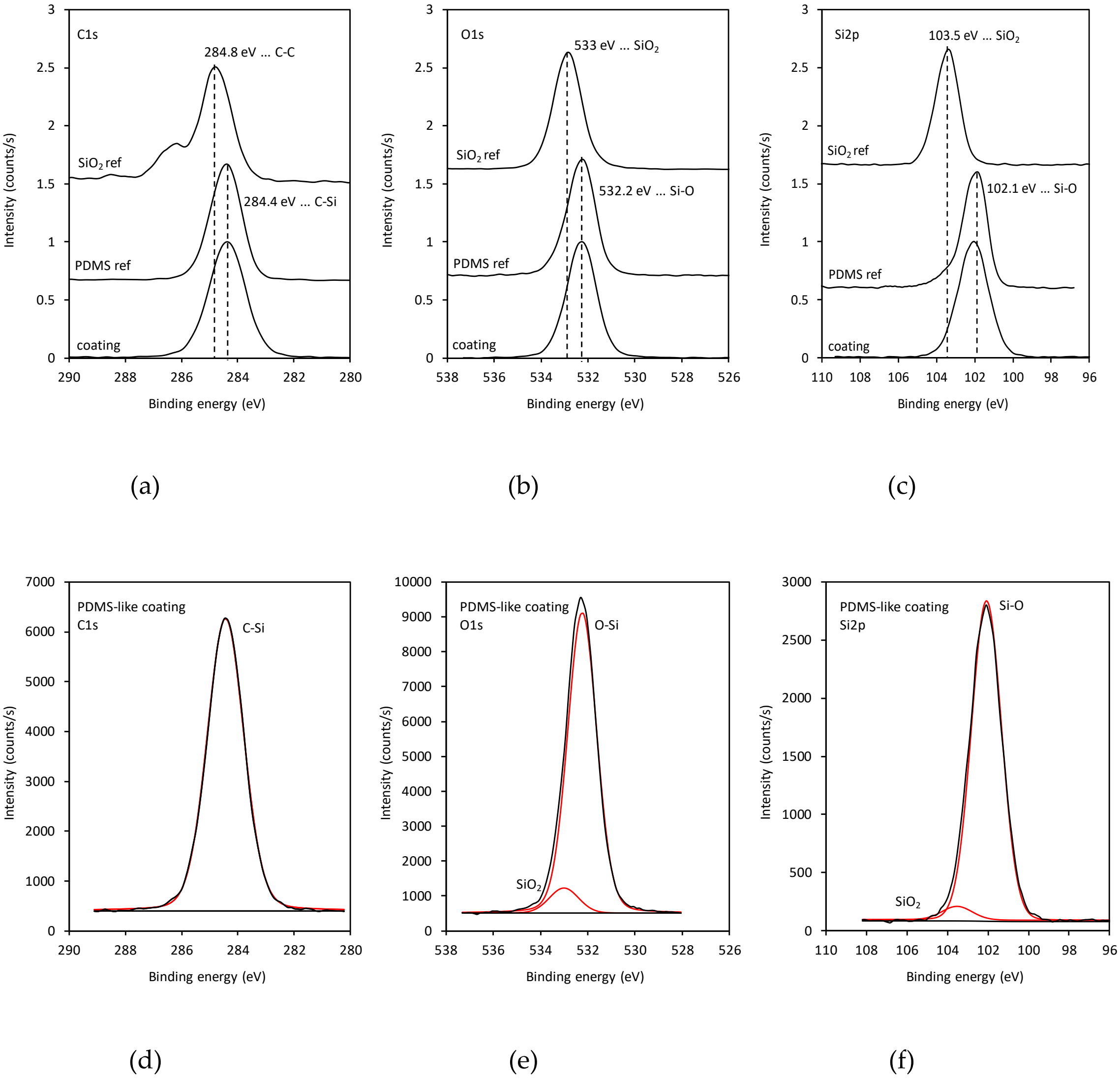

Figure S1. Comparison of XPS spectra of the coating with the reference samples of PDMS and SiO_2 for (**a**) C1s, (**b**) O1s, (**c**) Si2p and detailed surface spectra of the coating with subcomponents for (**d**) C1s, (**e**) O1s and (**f**) Si2p.
